# The German version of the self-efficacy questionnaire (SE-12-G) for measuring clinical communication skills in a sample of healthcare professionals: translation and psychometric properties

**DOI:** 10.1186/s12909-025-07681-y

**Published:** 2025-07-17

**Authors:** Wiebke Frerichs, Lene Marie Johannsen, Laura Inhestern, Corinna Bergelt

**Affiliations:** 1 Department of Medical Psychology, University Medical Center Hamburg-Eppendorf (UKE), Martinistraße 52, 20246 Hamburg, Germany; 2 Department of Medical Psychology, University Medicine Greifswald, Greifswald, Germany

**Keywords:** Psychometrics, Self-efficacy, Communication, Healthcare professionals, Medical education, Cancer, Oncology, Communication skill training, Family-centered care

## Abstract

**Background:**

Effective and patient-oriented communication is essential for healthcare professionals (HCPs) to deliver high quality care. Assessing communication skills training effectiveness relies on validated measures, such as the Self-Efficacy Questionnaire (SE-12). Yet, a validated German version is lacking. Thus, we aimed to translate and adapt the SE-12 into German and assess its psychometric properties.

**Methods:**

We translated the original SE-12 into German using the team translation protocol and adapted it to our context. We added a subscale on the importance of communication skills, resulting in the SE-12-G with two subscales (confidence and importance-scale). We conducted cognitive interviews with six HCPs to assess the comprehensibility of the SE-12-G. Afterwards, *n* = 152 HCPs completed the SE-12-G at two measurement time-points. We descriptively analyzed completion rate as indicator for acceptance, reliability (Cronbach’s α) and item characteristics (i.e., item difficulty, corrected item-total correlations, inter-item correlations). A confirmatory factor analysis was performed including three a priori hypothesized models including one that represents the factor structure performed by the authors of the original SE-12.

**Results:**

Cognitive interviews indicated that the translation and comprehensibility assessment of the German version of the SE-12 (SE-12-G) showed high face and content validity. Completion rate exceeded 98% for all items. Mean item difficulty across all items and subscales was 0.79, (ranging between 0.71 and 0.97), inter-item correlations ranged between 0.02 and 0.73 for the confidence-scale and between 0.044 and 0.533 for the importance-scale. Ceiling effects were present for half of the items on the confidence-scale, and for all items on the importance-scale. Internal consistency yielded a Cronbach’s α of 0.88 for the confidence-scale and 0.83 for the importance-scale. Confirmatory factor analysis supported a one-factor structure for both subscales of the SE-12-G.

**Conclusion:**

The translated and adapted German version of the SE-12 shows good levels of acceptance and reliability. Similarly to the original version, we found high ceiling effects in some items. Compared to the original factor structure, a one-factor structure was identified for both subscales in our sample. The SE-12-G should be evaluated in further studies and modification of some items should be considered.

**Supplementary Information:**

The online version contains supplementary material available at 10.1186/s12909-025-07681-y.

## Introduction

Effective and patient-oriented communication is essential for healthcare professionals (HCPs) to deliver high quality care to patients and their relatives [1, 2]. Good patient-provider communication can foster trust and rapport [3–5] (and can also improve patient-relevant outcomes (e.g. satisfaction [6], health [7], understanding [8, 9], stress [10]). Moreover, it can reduce HCPs’ stress experience and emotional burnout and increase job satisfaction [1, 8]. Reviews show that HCPs can be trained to increase their communication competencies through communication skills trainings in different clinical settings [1, 11, 12]. One central evaluation outcome of communication skills trainings is HCPs’ self-efficacy in communication skills [12–14]. In context of healthcare communication, self-efficacy can be defined as one’s belief in one’s own communication skills to handle certain situations when communicating with patients and/or their relatives.

Despite the importance of HCPs’ self-efficacy for the evaluation of communication skills trainings [12], only a few validated self-reported measures exist [15]. The majority of measures relies on self-constructed questionnaires with limited validity and reliability [12].

A short and specific measure of self-efficacy, that is applicable to various clinicians, is the self-efficacy questionnaire (SE-12) developed by Axboe et al. [15]. The SE-12 is available in Danish, English, Greek, Korean and Spanish [16–18] and measures self-efficacy of clinicians in their clinical communication skills. The original version is a 12-item, unidimensional instrument developed to assess self-reported self-efficacy in physicians and nurses in Denmark before and after participating in a communication skills training. The 12 items represent the key communications skill components of the Calgary Cambridge Guide [19]. Axboe and colleagues [15] found the SE-12 to be both comprehensive and comprehensible and a reliable and partly valid instrument, to assess the self-efficacy of clinical communication skills. The original SE-12 has been psychometrically tested by Axboe and collegues, revealing high internal consistency with a Cronbach’s α of 0.95 (range, 0.94–0.95) and acceptable test-retest reliability for the complete scale, with an intra-class correlation coefficient agreement (ICC) of 0.71 (range, 0.66–0.77), with ceiling effects evident in 9 of the 12 items [15]. Since its development, the SE-12 has been used, translated or been adapted for various studies [16, 20–22]. Two of these studies added an additional scale to assess the perceived importance of each item [19, 20]. Currently, there is no psychometrically tested instrument in German to assess HCPs’ self-efficacy in communicating with patients.

Therefore, we translated and adapted the original SE-12 scales into German and investigated the psychometric properties of the translated German version (SE-12-G) in a sample of German HCPs.

## Methods

### Study design and setting

The study is a secondary analysis of data collected within a three armed randomized controlled pilot-study conducted at the Department of Medical Psychology, University Medical Center Hamburg-Eppendorf (UKE), Germany between September 2019 and April 2021. The original study aimed to enhance the communication of HCPs working in oncology in the context of parental cancer involving *n* = 152 HCPs from Germany from outpatient and inpatient settings (e.g. hospitals, outpatient clinics, counseling centers [23, 24]). Participants were stratified by their profession and randomly assigned to three study groups (face-to-face training vs. e-Learning vs. waitlist-control group). Data collection included paper pencil questionnaires at three time points: before (t0, baseline before randomization), after (t1, post-training) and at 3-month follow-up (t2), assessing primary (competency to approach child- and family-related themes) and secondary outcomes (i.e. knowledge, communication behavior). The original study was registered within the German Clinical Trial Register (DRKS-00015794) and approved by the Local Psychological Ethics Committee of the Center for Psychosocial Medicine, UKE, Germany (LPEK‐001).

### The original SE-12 measure

The SE-12 measures self-efficacy of clinicians in their clinical communication [15]. The original version is a unidimensional instrument with 12 items using a 10-point response scale (1: very uncertain, 10: very certain) to measure confidence in their communication skills including an additional „not relevant“ check box [15]. Each question starts with the words “How certain are you that you are able to successfully…” followed by a specific communication skill [15]. Sum score of all 12 items is calculated, with higher scores indicating higher self-efficacy of clinical communication skills. The SE-12 has been adapted by adding a further scale measuring the perceived importance of each item by including a 5-point-scale (1: not important at all to 5: very important) [19, 20], so far without reporting on the psychometric properties of this additional scale.

### Translation and adaptation of the SE-12-G

In order to assess self-efficacy of HCPs in their communication competencies, the original SE-12 questionnaire [15] was translated into German following the TRAPD team translation protocol [25]. The TRAPD (Translation, Review, Adjudication, Pretesting and Documentation) method has been endorsed within the Guideline for Best Practice in Cross-Cultural Surveys [26] complying with best practice translation research [27]. An Excel spreadsheet was developed to assist with the translation and review process for each member of the research team and to document the process. Two team members (WF having a bilingual health science and physical therapy background, MLN a psychology background) independently translated the English version of the SE-12. A third blinded team member (CB) with experience in survey translation reviewed these translations, selecting one version or creating a new one as needed. Finally, all authors discussed the translations, until a consensus on a final version of the SE-12-G was reached. As a next step, the newly developed SE-12-G version was pretested by conducting cognitive interviews to assess comprehensibility and feasibility of the measure. Additionally, we adapted it by including a second scale, the perceived importance-scale, rating the same 12 items on a 5-point-Likert-scale (‘How important is it to incorporate this skill into your clinical work? 1: not important at all to 5: very important). The check box ‘not relevant’ from the original SE-12 scale was then added for both subscales altogether (see Table 3 for the English items of the SE-12-G and Additional file 1 for the German SE-12-G).

In the context of the previously described original randomized controlled pilot-study [23], cognitive interviews about the applied series of measures were conducted with various HCPs to assess the comprehensibility and feasibility in the context of content validity. Participants were recruited through convenience sampling from the authors’ professional contacts and networks, choosing individuals with relevant clinical experience (i.e., related to their work with oncology patients due to the nature of the original pilot-study or working in either in- or outpatient settings) to ensure they could effectively evaluate the comprehensibility and feasibility of the measures. During the cognitive interviews the think-aloud method was combined with the verbal probing technique [28] to review opinions on certain phrases, distinct words and their meanings for various HCPs and certain skills in daily practice (e.g., comprehensive probing “What does the term ‘non-verbal behavior’ mean to you?” or selection probing “When would you choose the answer ‘not relevant’”?). Interviews were audio-recorded and supported by handwritten protocols. After the interviews comments and suggestions from the material were discussed (WF, LJ, LI, CB). However, no further adaptations to the SE-12-G were necessary.

### Psychometric assessment

#### Participants and data collection

As this study is a secondary analysis of data from a randomized controlled pilot-study [23, 24], study participants were part of a sample of various HCPs in Germany participating in this pilot- study. HCPs were included if they were working in oncology (independent of setting, profession or amount of professional experience in oncology) [23]. They were recruited by e-mail or mail through existing networks, clinics and lists of cooperation partners. Participants did not receive any incentives for participation and participation was voluntary. In the original pilot-study, HCPs received a paper & pencil baseline questionnaire including the SE-12-G and were randomized by group stratification after returning the baseline questionnaire. The post-training questionnaire (t1) was sent after training and in the waitlist-control group 6 weeks after the return of the baseline questionnaire.

The SE-12-G was applied in a series of measures to assess HCPs’ competencies regarding child- and family-related issues in cancer care, including demographic variables (e.g., gender, age, profession, work experience with cancer patients) and experience with previous communication skills trainings were assessed. The SE-12-G was placed approximately two-thirds of the way through the paper-pencil questionnaire, following socio-demographic questions, the primary outcome measures, and knowledge items, with the entire questionnaire taking about 30 min to complete.

Data collection started in September 2019 and ended in April 2021. Data were entered into SPSS (IBM SPSS Statistics, V.27) including blinded double entry of 20% for quality control.

#### Data analyses

Descriptive statistics using frequencies for categorical data or mean values and standard deviation (SD) for metric data were calculated to describe the sample. In the following data analysis strategies will be reported, where Table 1 gives a detailed overview on established criteria used to interpret performed data analyses as well as data collection points.

Item analysis was performed for each subscale including calculation of item means and standard deviations (SD), corrected item-total correlations and inter-item correlations. Observed floor and ceiling effects were assessed by analyzing the distribution of participants marking the highest as well as lowest possible score per item and scale. The maximum score was 10 for the confidence-scale and 5 for the importance-scale. Item difficulty was assessed for each of the 12 items on both subscales. Internal consistency was estimated by calculating Cronbach’s coefficient alpha for each scale (confidence- and importance-scale). Test-retest reliability was assessed using the data of two measurement points from participants from waitlist-control group only, calculating Spearman correlation coefficient at item level for each of the 12 self-efficacy items for each scale, and calculating Pearson correlation for the sum score of the subscales. Content validity was assessed by exploring whether the SE-12-G reflects on characteristics of participants, namely “working experience in general” and “working experience with cancer patients” (both in years), similar to Axboe et al. [15]. For this purpose, we tested the relations of the confidence-scale and importance-scale to these two self-developed items by using bivariate correlations. To assess responsiveness to change, Cohen’s d was used to assess change in both intervention groups, analyzing the effect size of the intervention, complemented by an additional t-test to determine significance. For discriminant validity, known-group differences (Kruskal-Wallis test) comparing the four HCP groups at baseline were calculated followed by post-hoc tests using the Dunn’s test with Bonferroni correction for multiple comparisons. For these analyses, various data were used: for the responsiveness to change analysis, data from both intervention groups for the two measurement points (baseline and post-training questionnaire) were used; for the test-retest reliability, data from the waitlist-control group at two measurement points (baseline and 6-weeks after baseline) were used; for all other analysis only the baseline data was applied; the completion rate was calculated to assess the acceptance of the measure; the frequencies of missing data were calculated per item as well as for the overall measure and all cases; for all other calculations, missing data were replaced with item means. Cases were excluded if more than 30% of the SE-12-G items were missing [29].

To evaluate the factorial validity of the SE-12-G, we conducted a confirmatory factor analysis (CFA) [30–32]. To test assumptions for performing a factor analysis, Kaiser-Meyer-Olkin (KMO) measure of sampling adequacy and Bartlett’s test of sphericity were calculated [33, 34]. Three a priori models were hypothesized: Model 1, replicating the one-dimensional factorial structure of the original SE-12 measure for the confidence-scale only (M1); Model 2, assuming a two-factor structure with variables from both subscales (confidence and importance) treated as continuous variables and accounting for residual covariance between each item and scales to address methodological variance (M2); and model 3, an ordinal factor model (M3a) assuming a two-factor structure with both subscales, treating importance variables as ordinal due to the non-normal distribution of the data. The third model was further fitted to check modified indices and possible correlations between items on content-level (M3b) (e.g., item 3 (… *to encourage a patient to express and discuss their problems and concerns.*) and item 5 (… *to express their thoughts and feelings.*)).

For M1 and M2 full information maximum likelihood estimates were applied [35], for M3 (3a and 3b), the diagonally weighted least squares method, which is specifically designed for ordinal-scaled data with robust estimation [36], was performed. Kaiser-Meyer-Olkin (KMO) measure of sampling adequacy with values > 0.05 and the Bartlett’s test of sphericity with values < 0.05 were conducted to test criteria for calculating a CFA [33, 34].

To evaluate the global fit indices, the following were included: (a) the comparative fit index (CFI) and the Tucker-Lewis index (TLI); (b) the root mean square error of approximation (RMSEA), and the standardized root mean square residual (SRMR); and (c) average variance extracted (AVE) [33, 37, 38]. Further, factor covariance was explored to understand the relationship between the two latent factors confidence in and importance of these 12 communication skills.

Analysis of demographic data, analysis of completion rate, item analysis was performed using SPSS (IBM SPSS Statistics, Version 29.0.1.0). CFA and model of fit indices calculations were performed using JASP (JASP Team, 2023 Version 0.18.1), an open-source software, whose analyses are written in R.

**Table 1:** Psychometric analyses performed

**Psychometric analyses**	**Criteria/Description**	**Data collection (time points)**
Item Response Distributions	Evaluates the distribution of responses for each item to identify patterns or irregularities.	t0
Inter-Item Correlations	Assesses the degree of relationship between pairs of items. Values too close to 1 may indicate redundancy; too low values suggest lack of coherence. High inter-item correlations of above 0.80 indicate that items ask the same questions and might be redundant [33, 39].	t0
Corrected Item-Total Correlation	Measures the relationship between each item and the total score excluding the item. Higher values indicate good item consistency. Generally, values > 0.3 are considered acceptable, representing items measuring the same underlying concepts. Items with < 0.30 should be considered to be removed [40].	t0
Floor and Ceiling Effects	Identifies items where a significant portion of respondents scored the lowest (floor) or highest (ceiling) possible value. Cut-off values of > 15% of respondents are considered to be high [41].	t0
Item Difficulties	Quantifies the proportion of correct responses to an item. Ranges from 0 (most difficult) to 1 (easiest). Item difficulties are calculated by dividing item means by the maximal value of the answer range (0–4) and multiplying it with 100. Item difficulty should be near to 50%, and items should not differ much in their difficulty level [33, 34, 42].	t0
Cronbach’s α	Assesses internal consistency of the sub-scales, indicating the degree to which the items reliably measure the same underlying construct; values ≥ 0.70 are considered acceptable for early stages of research, with ≥ 0.80 preferred for established scales [40].	t0
Test-retest reliability	Assesses the stability of an instrument over time through repeated testing at two different time points; Spearman correlation coefficients at item level and Pearson correlation coefficients for subscale sum scores are used to evaluate temporal stability, with higher values indicating greater reliability—values close to zero suggest low reliability [40].	t0, t1^†^
Responsiveness to change	Assesses if the instrument detects meaningful changes over time in the construct it measures; Cohen’s d effect sizes indicate the magnitude of change, with values of 0.2 considered small, 0.5 moderate, and 0.8 large, and complemented by significance testing through t-tests.	t0, t1^††^
Discriminant validity	Assesses the instrument’s ability to distinguish between groups expected to differ (also known as known-group differences), using statistical tests (Kruskal-Wallis test), followed by post-hoc analyses (Dunn’s test with Bonferroni correction for multiple comparisons). Statistically significant results indicate which group differs in respect to the variable measured.	t0
Kaiser-Meyer-Olkin (KMO) Measure	Values > 0.6 suggest adequate sample size for factor analysis [33].	t0
Bartlett’s Test of Sphericity	Tests the hypothesis that the correlation matrix is an identity matrix, indicating suitability for factor analysis. *p* < 0.05 indicates appropriateness [33].	t0
Comparative Fit Index (CFI)	CFIs is an indicator for model fit. It ranges from 0 to 1 and higher values indicate better fit. Values above 0.95 indicate a good model fit [37, 43].	t0
Tucker-Lewis Index (TLI)	Values > 0.90 suggest a good fit of the model to the data in confirmatory factor analysis [37].	t0
Root Mean Square Error of Approximation (RMSEA)	Values ≤ 0.05 indicate a close fit, values up to 0.08 represent reasonable errors of approximation in the population [44].	t0
Standardized Root Mean Square Residual (SRMR)	Values ≤ 0.08 are generally considered good, indicating small residuals between observed and model-implied covariances [37].	t0
Average Variance Extracted (AVE)	Represents the average of the squared factor loadings for all observed variables associated with a particular construct with recommended values of ≥ 0.5 [45, 46].	t0

Notes: † waitlist control group only; ^††^ intervention groups face-to-face and e-learning only

## Results

### Translation and adaptation of the SE-12-G

Both translators (WF and MLN) and the reviewer (CB) translations were largely consistent, with only minor differences in the wording observed in terminology of specific items and the response scale as well as the structure of the sentence. Identified differences were for found for the introduction (how to best translate the word “skills”) as well as single items, namely item 2 (agenda vs. themes), item 7 (adding e.g., to the examples), item 8 (by using a German synonym for empathy), item 11 (the word “shared” was translated differently) and item 12 (the word “assuring” was translated differently). Additionally, the response format word for “not relevant” differed between both translators. In summary, only the choice of single words differed between the translators without differences in meaning (e.g. the word “patient” also represents the word “client”, which is often used in psycho-oncology, psycho-social and psychotherapy settings). Within the first round of team discussion (adjudication process) all authors reached consensus on a final version.

To test the German version of the SE-12 for comprehensibility, cognitive interviews with *n* = 6 HCPs (*n* = 2 physicians, *n* = 2 nurses, *n* = 2 psychologists; 50% being female) were conducted by assessing the complete evaluation tool developed for the original pilot-study [23]. Cognitive interviews for the complete 13-page baseline questionnaire including a series of measurements lasted for about 75 min each. As participants had no critical feedback or comprehensibility issues on the instructions and items of the SE-12-G, no further adaptions to the final version were necessary and thus no further interviews were planned with additional HCPs. The final SE-12-G measure used in this study can be found in the Additional file 1, the English version is displayed in Table 3.

### Psychometric assessment

#### Sample characteristics

Data of *n* = 152 participants were included in this secondary data analysis. The mean age was 44.4 years (SD 11.6) with 88% being female. Most of the participants were psychologists (37.5%), followed by physicians (26.3%), nurses (18.4%) and social workers/others (17.8%). Of their professional experience working with cancer patients, most had > 11 years of experience (39.1%), followed by 1–5 years (30.5%), 6–10 years (21.9%) and 8.6% less than 1 year (see Table 2).

**Table 2:** Sample characteristics (*n* = 152)

	Total^†^ (*n* = 152)
**Age**, M (SD) [range]	44.42 (11.6) [24–71]
**Sex**, n (%)	
female	134 (88.2)
male	18 (11.8)
**Professional group**, n (%)	
Physician	40 (26.3)
Nurse	28 (18.4)
Psychologist	57 (37.5)
Social Worker/Other	27 (17.8)
**Workplace setting**^**†**^, n (%)	
outpatient	94 (61.8)
inpatient	84 (55.3)
self-employed/registered	25 (16.4)
other	8 (5.3)
**Working experience with cancer patients**, n (%)	
< 1 year	13 (8.6)
1–5 years	46 (30.5)
6–10 years	33 (21.9)
> 11 years	59 (39.1)
**Amount of cancer patients per month**, No. M (SD) [range], %	67.4 (33.0) [0.1-100], *n* = 143
**Amount of cancer patients between 25–55 years old**, M (SD) [range] in %	37.52 (22.21) [0.1-100], *n* = 131
**Amount of cancer patients parenting minor children**, M (SD) [range] in %	21.4 (21.9) [0-100], *n* = 123
**Having participated in communication skills trainings before**, n (%)	134 (88.2)

Abbreviations: M: Mean, SD: Standard Deviation

Notes: ^†^ sample sizes slightly differ, as single items were not answered by all respondents. ^††^Multiple answers possible

#### SE-12-G item analysis

Table  3 shows response distribution, distribution of participants with highest score (floor effects), acceptance, corrected item-total correlation and item difficulty of the 12 items.

Depending on the item, between 5.3% and 6.6% of the participants rated the respective item as “not relevant”. Missing values ranged from 1.3 to 2% per item. Considering all items, completion rate was over 98% of the SE-12-G.

Ceiling effects were present, for the confidence-scale in 6 of the 12 items (items 3, 4, 5, 7, 8, 10) with a range from 15.8% (item 10) to 30.3% (item 8) of respondents, exceeding the > 15% set as limit [41] and therefore indicating high ceiling effects. Regarding the importance-scale, all 12 items present ceiling effects with a range from 54.6% (item 9 and 11) to 85.5% (item 8).

Corrected item-total correlation values for the confidence-scale ranged from 0.46 (item 4) to 0.66 (item 3), for the importance-scale values ranged from 0.34 (item 1) to 0.62 (item 10) with Items 3 for the confidence-scale and item 10 for the importance-scale suggesting stronger relationships between the items and the construct. In contrast, item 4 for the confidence-scale and item 1 for the importance-scale indicate weaker associations with the construct with correlation values of < 0.30.

The mean item difficulty (Table 3 ) across all items was 0.79 for the confidence-scale (range 0.71–0.87) and 0.91 for the importance-scale (range 0.84–0.97) indicating that, on average, items tended to be relatively easy for participants.

Inter-item correlations (Table 4) for the confidence-scale ranged from 0.019 (item 4 and item 11) to 0.733 (item 3 and item 5), for the importance-scale from 0.044 (item 5 and item 8) to 0.533 (item 2 and item 10), indicating that item 4 and 11 lack coherence.

**Table 3:** Descriptive statistics of participants for the SE-12-G, response distribution, means, standard deviation, acceptance, item discrimination and item difficulty (n=139-149 healthcare professionals)

		N	M (SD), [Min, Max]	% participants with highest score	Acceptance (number of missings, n (%))	Relevance (number of ‘not relevant’, n (%))	Item discrimination (corrected item-total correlation)	Item difficulty (mean)
How certain are you that you are able to successfully… / How important is it to…	Scale							
1.…identify the issues the patient wishes to address during the conversation?	C-Scale	149	7.83 (1.36), [4, 10]	8.6	2 (1.3)	1 (.7)	0.61	0.76
	I-Scale	148	4.84 (.47), [1, 5]	83.6	3 (2)		0.34	0.96
2.… make an agenda/plan for the conversation with the patient?	C-Scale	142	7.81 (1.48), [3, 10]	10.5	2 (1.3)	8 (5.3)	0.57	0.76
	I-Scale	141	4.55 (.6), [3, 5]	55.9	3 (2)		0.59	0.89
3.… urge the patient to expand his or her problems/worries?	C-Scale	148	8.51 (1.39), [4, 10]	27	3 (2)	1 (.7)	0.66	0.83
	I-Scale	149	4.78 (.46), [3, 5]	78.3	3 (2)		0.45	0.94
4.… listen attentively to the patient?	C-Scale	149	8.84 (1.28), [5, 10]	38.8	2 (1.3)	1 (.7)	0.46	0.87
	I-Scale	149	4.77 (.46), [3, 5]	76.3	2 (1.3)		0.48	0.94
5.… encourage the patient to express thoughts and feelings?	C-Scale	147	8.36 (1.34), [5, 10]	22.4	2 (1.3)	3 (2)	0.63	0.82
	I-Scale	146	4.69 (.53), [3, 5]	69.7	2 (1.3)		0.47	0.92
6.… structure the conversation with the patient?	C-Scale	146	7.44 (1.63), [3, 10]	9.9	1 (.7)	5 (3.3)	0.62	0.72
	I-Scale	146	4.35 (.72), [2, 5]	46.1	1 (.7)		0.49	0.84
7.… demonstrate appropriate non-verbal behavior (eye contact, facial expression, placement, posture, and voicing)?	C-Scale	148	8.28 (1.46), [2, 10]	17.8	2 (1.3)	2 (1.3)	0.50	0.81
	I-Scale	148	4.68 (.51), [3, 5]	68.4	2 (1.3)		0.42	0.92
8.… show empathy (acknowledge the patient's views and feelings)?	C-Scale	148	8.85 (1.02), [6, 10]	30.3	2 (1.3)	2 (1.3)	0.52	0.87
	I-Scale	148	4.87 (.36), [3, 5]	85.5	2 (1.3)		0.55	0.97
9.… clarify what the patient knows in order to communicate the right amount of information?	C-Scale	145	7.81 (1.55), [2, 10]	11.2	2 (1.3)	5 (3.3)	0.59	0.76
	I-Scale	145	4.52 (.60), [3, 5]	54.6	2 (1.3)		0.46	0.88
10.… check patient's understanding of information given?	C-Scale	144	8.31 (1.50), [2, 10]	15.8	2 (1.3)	5 (3.3)	0.57	0.79
	I-Scale	145	4.68 (.54), [3, 5]	67.8	2 (1.3)		0.62	0.92
11.… make a plan based on shared decisions between you and the patient?	C-Scale	139	7.76 (1.69), [2, 10]	13.2	3 (2)	10 (6.6)	0.55	0.75
	I-Scale	139	4.52 (.65), [2, 5]	54.6	3 (2)		0.42	0.88
12.… close the conversation by assuring, that the patient's questions have been answered?	C-Scale	148	7.70 (1.67), [2, 10]	11.2	2 (1.3)	2 (1.3)	0.56	0.75
	I-Scale	149	4.58 (.64), [2, 5]	63.8	2 (1.3)		0.50	0.90
Overall Confidence-Scale (Items 1–12)			8.11 (.95), [5, 10]	18.06	n/a	n/a	n/a	0.79
Overall Importance-Scale (Items 1–12)			4.65 (.33), [3.67, 5]	67.05	n/a	n/a	n/a	0.91

Abbreviations. M: Mean, SD: Standard Deviation; C-Scale: Confidence-scale (range 1-10; 1: very uncertain to 10: very certain); I-Scale: Importance-scale (range 1-5;; 1: not important at all to 5: very important)

**Table 4:** Inter-Item Correlation for the SE-12-G (n=131-132 healthcare professionals)

Confidence-scale													
	Item 1	Item 2	Item 3	Item 4	Item 5	Item 6	Item 7	Item 8	Item 9	Item 10	Item 11	Item 12	
**Item 1**	1	0,541	0,679	0,436	0,552	0,403	0,351	0,508	0,329	0,209	0,262	0,248	
**Item 2**	0,541	1	0,451	0,334	0,422	0,525	0,228	0,35	0,264	0,366	0,326	0,312	
**Item 3**	0,679	0,451	1	0,473	0,733	0,392	0,325	0,573	0,315	0,258	0,299	0,359	
**Item 4**	0,436	0,334	0,473	1	0,596	0,279	0,347	0,456	0,17	0,144	0,019	0,288	
**Item 5**	0,552	0,422	0,733	0,596	1	0,368	0,3	0,462	0,308	0,336	0,253	0,295	
**Item 6**	0,403	0,525	0,392	0,279	0,368	1	0,278	0,308	0,512	0,446	0,473	0,399	
**Item 7**	0,351	0,228	0,325	0,347	0,3	0,278	1	0,381	0,388	0,431	0,338	0,332	
**Item 8**	0,508	0,35	0,573	0,456	0,462	0,308	0,381	1	0,151	0,203	0,157	0,313	
**Item 9**	0,329	0,264	0,315	0,17	0,308	0,512	0,388	0,151	1	0,61	0,602	0,464	
**Item 10**	0,209	0,366	0,258	0,144	0,336	0,446	0,431	0,203	0,61	1	0,533	0,413	
**Item 11**	0,262	0,326	0,299	0,019	0,253	0,473	0,338	0,157	0,602	0,533	1	0,562	
**Item 12**	0,248	0,312	0,359	0,288	0,295	0,399	0,332	0,313	0,464	0,413	0,562	1	
**Importance-scale**													
	**Item 1**	**Item 2**	**Item 3**	**Item 4**	**Item 5**	**Item 6**	**Item 7**	**Item 8**	**Item 9**	**Item 10**	**Item 11**	**Item 12**	
**Item 1**	1	0,356	0,193	0,252	0,257	0,157	0,147	0,263	0,157	0,247	0,135	0,142	
**Item 2**	0,356	1	0,442	0,373	0,425	0,367	0,264	0,365	0,238	0,523	0,275	0,232	
**Item 3**	0,193	0,442	1	0,227	0,521	0,289	0,17	0,361	0,152	0,331	0,07	0,277	
**Item 4**	0,252	0,373	0,227	1	0,376	0,24	0,224	0,38	0,282	0,306	0,168	0,323	
**Item 5**	0,257	0,425	0,521	0,376	1	0,33	0,253	0,423	0,044	0,272	0,072	0,268	
**Item 6**	0,157	0,367	0,289	0,24	0,33	1	0,353	0,339	0,23	0,363	0,253	0,293	
**Item 7**	0,147	0,264	0,17	0,224	0,253	0,353	1	0,373	0,23	0,278	0,217	0,211	
**Item 8**	0,263	0,365	0,361	0,38	0,423	0,339	0,373	1	0,289	0,374	0,156	0,304	
**Item 9**	0,157	0,238	0,152	0,282	0,044	0,23	0,23	0,289	1	0,432	0,499	0,398	
**Item 10**	0,247	0,523	0,331	0,306	0,272	0,363	0,278	0,374	0,432	1	0,378	0,407	
**Item 11**	0,135	0,275	0,07	0,168	0,072	0,253	0,217	0,156	0,499	0,378	1	0,385	
**Item 12**	0,142	0,232	0,277	0,323	0,268	0,293	0,211	0,304	0,398	0,407	0,385	1	

#### Internal consistency and test-retest reliability

Cronbach’s alpha coefficient for SE-12-G was high with a calculated α of 0.88 for the confidence-scale and α 0.83 for the importance-scale.

The test-retest reliability at item level for the two subscales was good with estimated correlation coefficient for the 12 items for the confidence-scale being *r* = 0.725 with a 95% Bootstrap Confidence Interval (BCI) of 0.484–0.874 and for the importance-scale estimated correlations of *r* = 0.726 with a 95% BCI of 0.479–0.862 (Table 5). For the confidence-scale no item showed a weak correlation, 5 items showed a moderate correlation of 0.430–0.491 (item 2, 3, 7, 11, 12) and 6 items showed strong correlations ranging from 0.538 to 0.755 (item 1, 4, 5, 6, 8, 9, 10). For the importance-scale, two items had a strong correlation (item 4 *r* = 0.605, item 5 *r* = 0.632), 8 items a moderate correlation ranging from 0.327 to 0.421 (item 1, 2, 3, 7, 8, 9, 10, 12) and two items a weak correlation (item 6 *r* = 0.160, item 11 *r* = 0.226).

**Table 5:** Test-retest reliability (n=40-74 healthcare professionals)

How certain are you that you are able to successfully… / How important is it to…	Scale	N	*r*	95% CI
1.…identify the issues the patient wishes to address during the conversation?	C-Scale	47	0.755**	.540;-.863
	I-Scale	47	0.415**	.053;-.703
2.… make an agenda/plan for the conversation with the patient?	C-Scale	44	0.481**	.154;-.719
	I-Scale	44	0.351*	.027;-.642
3.… urge the patient to expand his or her problems/worries?	C-Scale	46	0.443**	.125;-.700
	I-Scale	46	0.381**	.017;-.681
4.… listen attentively to the patient?	C-Scale	47	0.639**	.344;-.850
	I-Scale	47	0.605**	.342;-.831
5.… encourage the patient to express thoughts and feelings?	C-Scale	45	0.699**	.480;-.833
	I-Scale	45	0.632**	.345;-.833
6.… structure the conversation with the patient?	C-Scale	47	0.538**	.269;-.730
	I-Scale	47	0.160	-.160;-.464
7.… demonstrate appropriate non-verbal behaviour (eye contact, facial expression, placement, posture, and voicing)?	C-Scale	46	0.491**	.202;-.742
	I-Scale	46	0.349*	.004;-.645
8.… show empathy (acknowledge the patient's views and feelings)?	C-Scale	46	0.659**	.439;-.819
	I-Scale	46	0.327*	-.092;-.697
	I-Scale	45	0.421**	.139;-.672
10.… check patient's understanding of information given?	C-Scale	45	0.556**	.332;-.726
	I-Scale	45	0.412**	.954;.707
11.… make a plan based on shared decisions between you and the patient?	C-Scale	41	0.481**	.131;-.753
	I-Scale	40	0.226	-.110;-.536
12.… close the conversation by assuring, that the patient's questions have been answered?	C-Scale	46	0.430**	.143;-.668
	I-Scale	46	0.417**	.128;-.700
Overall Confidence-Scale (Items 1–12)†		47	0.725**	.484;-.874
Overall Importance-Scale (Items 1–12)†		47	0.726**	.479;-.862

Abbreviations: C-Scale: Confidence-scale (range 1-10; 1: very uncertain to 10: very certain); I-Scale: Importance-scale (range 1-5;; 1: not important at all to 5: very important); The correlation (two-sided) is significant at *p < 0.05, ** p < 0.001; 95% Bootstrap Confidence Interval reported in brackets; †Pearson correlation, all other items were calculated with Spearman correlation coefficient

#### Convergent validity

Statistically significant but weak correlations were found between the confidence-scale and the items *working experience in general* (*r* = 0.203**, range 0.043–0.350) and *working experience with cancer patients* (*r* = 0.147**, range 0.085–0.406). No significant correlations were found for the importance-scale for *working experience in general* (*r* = 0.035, range − 0.116–0.191) and *working experience with cancer patients* (*r* = 0.147, range − 0.093–0.250).

#### Responsiveness to change

The Cohen’s d for the confidence-scale was 0.77 (95% CI [4.0, 5.5]), indicating a moderate to large effect size, suggesting responsiveness for change after an intervention (Table 6). For the importance-scale Cohen’s d was 0.25 (95% CI [-0.167, 0.275]) indicating a small effect, suggesting that this subscale shows limited sensitivity in detecting changes over time.

**Table 6:** Responsiveness to change

Scale	*N*†	Mdiff (SD) [95% CI]	T	df	*p*	d [CI 95%]
Confidence-scale t1-t0	80	3.68 (0.77) [3.5;3.8]	42.727	79	< 0.000	0.77 [4.0;5.5]
Importance-scale t1-t0	79	0.01 (0.25) [-0.4;0.07]	0.475	78	0.636	0.25 [-0.167;0.275]

Abbreviations: † Intervention participants only (face-to-face and E-Learning, two measurement points, baseline (t0) and after-training participation (t1); Mdiff, mean difference (t1-t0); SD, Standard Difference; CI, Confidence Interval; T, df, degrees of freedome; p, *p*-value; d, Cohen’s d effect

#### Discriminant validity

Analyses of group differences between HCP groups revealed statistically significant differences among the groups for several items of the confidence-scale (e.g., urge the patient to express his or her problems/worries, listen attentively to the patient, demonstrate appropriate non-verbal behavior, show empathy, make a plan based on shared decisions) and for two items in the importance subscale (listen attentively to the patient, encourage the patient to express thoughts and feelings). Most differences were between physicians and psychologists as well as nurses and psychologists with psychologists scoring higher in self-efficacy (Table 7).

**Table 7:** Discriminant validity - Known-group differences comparing the four HCP groups (n=93-98 HCPs)

Items		$\:{x}^{2}$ ^†^	PHY	NRS	PSY	SW/O	Mean difference *z* (SE) ^††^				
How certain are you that you are able to successfully… / How important is it to…	Scale		Mean rank	Mean rank	Mean rank	Mean rank	PHY vs NRS	PHY vs SW/O	PHY vs PSY	NRS vs PSY	NRS vs SW/O
1.…identify the issues the patient wishes to address during the conversation?	C-scale	9.630*	40.80	38.03	56.96	56.67	ns	ns	ns	ns	ns
	I-scale	3.597	45.80	46.94	52.95	46.94	ns	ns	ns	ns	ns
2.… make an agenda/plan for the conversation with the patient?	C-scale	6.381	43.84	34.40	53.53	51.19	ns	ns	ns	ns	ns
	I-scale	2.276	46.62	44.70	45.82	55.50	ns	ns	ns	ns	ns
3.… urge the patient to expand his or her problems/worries?	C-scale	11.602**	39.40	40.29	60.68	48.61	ns	ns	-21.284 (7.076)*	ns	ns
	I-scale	5.595	45.92	41.79	53.12	54.11	ns	ns	ns	ns	ns
4.… listen attentively to the patient?	C-scale	9.521	38.28	46.47	59.26	47.33	ns	ns	-20.983 (6.988)*	ns	ns
	I-scale	9.294*	41.34	59.00	48.64	53.67	-17.660 (6.134)*	ns	ns	ns	ns
5.… encourage the patient to express thoughts and feelings?	C-scale	5.893	43.48	40.62	57.18	50.03	ns	ns	ns	ns	ns
	I-scale	11.357**	42.21	38.88	57.00	50.72	ns	ns	ns	-18.118 (6.323)*	ns
6.… structure the conversation with the patient?	C-scale	1.807	48.38	39.83	50.88	48.21	ns	ns	ns	ns	ns
	I-scale	5.964	52.96	53.47	40.37	52.94	ns	ns	ns	ns	ns
7.… demonstrate appropriate non-verbal behaviour (eye contact, facial expression, placement, posture, and voicing)?	C-scale	10.728*	44.34	34.18	59.09	50.89	ns	ns	ns	-24.916 (8.061)*	ns
	I-scale	1.110	49.40	45.94	52.30	47.08	ns	ns	ns	ns	ns
8.… show empathy (acknowledge the patient's views and feelings)?	C-scale	9.688*	41.60	41.41	60.08	45.78	ns	ns	-18.479 (7.008)*	ns	ns
	C-scale	6.669	45.56	44.09	54.22	50.11	ns	ns	ns	ns	ns
9.… clarify what the patient knows in order to communicate the right amount of information?	I-scale	1.233	51.86	48.18	44.99	51.53	ns	ns	ns	ns	ns
	C-scale	2.516	54.90	47.85	45.99	45.21	ns	ns	ns	ns	ns
10.… check patient's understanding of information given?	I-scale	2.924	46.77	44.68	53.41	41.29	ns	ns	ns	ns	ns
	C-scale	1.938	48.33	42.94	47.51	53.65	ns	ns	ns	ns	ns
11.… make a plan based on shared decisions between you and the patient?	I-scale	9.990**	58.08	31.07	45.63	47.93	ns	ns	ns	27.013 (8.621)**	ns
	C-scale	2.267	52.74	42.40	45.26	46.43	ns	ns	ns	ns	ns
12.… close the conversation by assuring, that the patient's questions have been answered?	I-scale	4.886	47.14	38.50	55.92	49.61	ns	ns	ns	ns	ns
	C-scale	1.311	50.32	43.82	51.51	49.47	ns	ns	ns	ns	ns

PHY: Physician; NRS: Nurses; PSY: Psychologists; SW/O: Social Workers/Others; † Degrees of freedom = 3; †† Dunn's test with Bonferroni correction for multiple comparisons; *p < 0.05, ** *p* ≤ 0.01; ns, not significant ( *p* >0.05)

#### Factor analysis

**Fig. 1:**
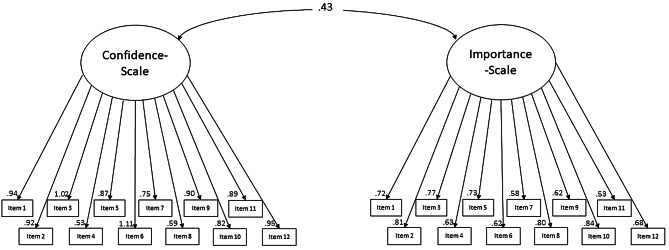
Confirmatory factor analysis model for one-factorial structure (model 3b)

Requirements for factor analysis were met, with an adequate sample size of *n* = 149 HCPs related to the indicators per factor [47], after three cases were excluded due to > 30% missing items. Additionally, further requirements were met, with KMO measure at 0.0.843 and Bartlett’s test of sphericity yielding X²=829.190, *p* < 0.001 [33, 34]. Model fit indices of the three a priori models are presented in Table 8. Factor reliability was satisfactory for all three models (M1: ω 0.827; M2: ω 0.891; M3a: ω 0.839 confidence and ω 0.882 importance), and factor loadings for all 12 items for both subscales were medium to high (M1: between 0.477 and 0.799 for the confidence-scale only; M2: 0.395–0.751 for both subscales; M3a: 0.405–0.810 for both subscales; M3b: 0.410–0.837)(see Fig. 1 for M3b and Additional file 2 for figures of M1-3a), indicating that the presented items (communication skills) measure the underlying construct (confidence in and importance of these skills). Factor covariance for M2 and M3a was 0.426 and for M3b 0.432, indicating a moderate correlation between the two factors, affirming their distinctness as separate constructs.

**Table 8:** Fit indices for the SE-12-G questionnaire

Model	χ2	df	*P* value	CFI	TLI	RMSEA [90% CI]	SRMR	AVE
M1	268.346	54	< 0.001	0.712	0.648	0.163	0.106	0.364
M2	579.761	239	< 0.001	0.776	0.741	0.097	0.092	C: 0.396 I: 0.331
M3a	366.631	235	< 0.001	0.888	0.868	0.061	0.098	C: 0.357 I: 0.497
M3b	376.144	235	< 0.001	0.880	0.859	0.063	0.099	C: 0.359 I: 0.489

Note: M1 = Axboe et al. Model, only confidence-scale; M2 = The model which included confidence- and importance-scale for each 12 items applying continuous variables; M3a = The model which included confidence- and importance-scale for each 12 items, applying ordinal variables for the importance-scale; M3b = like M3a but additional modifications for assumed residual covariances for items 3 and 5 as well as 9 and 10

Abbreviations: AVE, average variance extracted; CFI, comparative fit index; CI, confidence interval; df, degrees of freedom; RMSEA, root mean square error of approximation; SRMR, standardized root mean square residual; TLI, Tucker–Lewis index

Regarding the model fit indices, values indicated a suboptimal fit of the three assumed models (Table 8). The convergent validity of all three models was low, with higher, but still inadequate AVE values for M3a and M3b for both factors (Table 8), indicating that the latent constructs do not adequately capture the variance in its observed indicators [38].

## Discussion

The present study aimed to translate the original SE-12 by Axboe et al. [15] into German and assess its psychometric properties. The SE-12-G instrument proved to be a comprehensible and reliable tool and showed acceptable validity despite moderate fit indices.

### Translation and assessment of comprehensibility as part of content validity

The translation process yielded a comprehensible and valid German version of the SE-12. Comprehensibility testing through cognitive interviews with HCPs confirmed the clear understanding of the questionnaire, leading to no further adaptations. Inclusion of more participants with various backgrounds (e.g., various migration and cultural backgrounds) for testing the face-validity of the SE-12-G would provide information on the need of further adaptations.

### Analyses of SE-12-G items, reliability and validity

The item analysis revealed generally high response rates and a low rate of missing values, indicating good participant engagement and the SE-12-G to be well-accepted by various HCPs.

Corrected item-total correlations are mostly above 0.66, indicating that most items measure the same underlying concept. Inter-item correlations are all above 0.30 indicating that all items are relevant and none should be deleted [40]. Criteria for good item difficulties are met as mean item difficulty was 0.79 for the confidence-scale and for the importance-scale 0.91 and all values are above 50% highlighting the overall ease in responding [34]. However, multiple ceiling effects were present. This is in line with previous studies applying the original SE-12 confidence-scale only [15, 18, 19], indicating that especially the importance-scale might be redundant. High ceiling effects may impede the identification of potential training effects in evaluations of communication skills trainings for HCPs. Nevertheless, the evaluation study by Johannsen et al. [23] found statistically significant improvements in the confidence and importance scores of the SE-12-G after training participation compared to the waitlist-control group at post-training assessment, supporting the SE-12-G sensitivity to detect change over time. As our sample comprises a large proportion of psychosocial HCPs and participation in a communication skills training was motivation for participation, further application of the SE-12-G in mixed samples of HCPs should be conducted to assess ceiling effects, especially regarding the importance-scale. Regarding the internal consistency, the SE-12-G shows excellent values and an overall good test-retest reliability, which indicates that the questionnaire was consistent over the two measurement points [48, 49].

To our knowledge there are no measures that are related to the SE-12-G and could have been used to assess same or similar constructs. Therefore, we used latent constructs similar to Axboe and colleagues to explore the convergent validity of the SE-12-G. Our results show weak correlations between the confidence-scale and working experience (in general and with cancer patients), which is contrary to the findings of Axboe et al. [15]. However, subsequent studies including other measurements, e.g., Attitudes towards Medical Communication [18], should be conducted to assess convergent validity. Regarding the discriminant validity, results indicate that HCPs’ confidence (*d* = 0.77) and importance (d = 0.25) changed after a communication skills training. Known-group differences revealed various significant differences between different HCP subgroups in our sample in responses for specific items on the confidence-scale of the SE-12-G. Especially psychologists differed significantly and more often from physicians and nurses on the confidence-items. These findings highlight potential distinctions in perceptions among HCP groups regarding communication skills, which may be explained by different education, focus of care and time with the patients (e.g. psychologists and physicians).

### Factor validity

We a priori hypothesized three models for the SE-12-G: a one-factor model replicating the original structure (M1), a two-factor model with continuous variables and residual covariances to account for methodological variance (M2), and an ordinal two-factor model to address non-normal data (M3a), which was then fitted to check modified indicies and possible correlations (M3b) (similarities in content between items, e.g., item 3 and item 5, item 9 and 10, see additional file 2). A confirmatory factor analysis confirmed the one-factorial structure of the original SE-12 with the confidence-scale only (M1), but fit indices were of no good fit. We tested additional a priori models, which slightly improved the model fit (especially M3b), but still could not find acceptable values. As the convergent validity of all three models was low, with higher, but still inadequate AVE values for M3a and M3b for both factors (Table 8), values indicated that the latent constructs do not adequately capture the variance in its observed indicators [38]. Yet, employing AVE for convergent validity relies on rules of thumb rather than statistical testing procedures, neglecting sampling errors and limiting the generalizability of conclusions to broader populations [50]. Therefore, we prefer M3b (CFI = 0.880, TLI = 0.859, RMSEA = 0.063 (95%CI)), but recommend to explore the CFA again with (a) a larger and more heterogenic sample [51] and (b) additional modifications (e.g. the exclusion of items with high ceiling effects).

Additionally, after further analysis for the SE-12-G and discussions with external experts on psychometric evaluations, methodology adjustments of the importance-scale might be necessary, to decrease its methodological dependence to the confidence-scale (i.e. “How important do you think it is to implement *this* in everyday working life?“ regarding the term “this”.) Still, as factor covariance lies within the cut off values of 0.30 and 0.70, results indicate that these two scales are distinct constructs despite the methodological dependence regarding the wording.

### Limitations and strengths

This study has several limitations. First, divergent validity as part of psychometric parameters could not be analyzed due to the nature of this study being a secondary analysis. Second, the feasibility and comprehensibility testing of the SE-12-G was conducted with six HCPs only through convenience sampling. Additional cognitive interviews with a larger and broader range of HCPs could strengthen the feasibility and comprehensibility of the SE-12. Third, the SE-12-G was applied to a selective sample of HCPs participating voluntarily in a communication skills training on child- and family-specific themes, resulting in sampling and volunteer bias. Further validation in different settings without participation in a communication skills training is needed to ensure generalizability. Fourth, some items exhibit close content-related associations, potentially exerting an influence in the variance within the factor analysis. A careful revision of the translated items might be indicated for these items. Lastly, the SE-12-G was included within a series of measures to evaluate the effects of a communication skills training, positioned about two-thirds into the questionnaire, which in total took approximately 30 min and was answered after the knowledge questions, potentially affecting its scores.

A strength of this study is that we conducted an elaborated translation procedure, aligned with recommended survey translations. Additionally, we used cognitive interviews to explore face validity with various HCPs working in the field of communication and oncology. Further, we explored various models with an adequate sample to robustly perform factor analysis and various psychometric analyses.

## Conclusion

Self-efficacy of HCPs in their communication skills should be assessed with valid and reliable measures. So far, there has been no applicable and valid tool for all HCPs in Germany. With this study, we provide the first German measure for assessing self-efficacy in communication skills of HCPs including two subscales, the confidence- and importance-scale. The German SE-12 (SE-12-G) is a brief measure with good acceptance and reliability. In our sample, psychometric properties were limited regarding the factor analysis and ceiling effects. This could be due to the sample composition (e.g. voluntary bias). We suggest, that future research should modify the SE-12-G further (e.g. by excluding items with high ceiling effects) and evaluate the instrument in a larger and more heterogenious sample of HCPs, who are not taking part in an intervention. This should include checking for possible redundancy in the importance-scale and to include other instruments for assessing convergent validity.

## Electronic supplementary material

Below is the link to the electronic supplementary material.

Supplementary Material 1

Supplementary Material 2

## Data Availability

The dataset collected and analyzed during this study is available from the corresponding author on reasonable request and after consultation of the Local Psychological Ethics Committee of the Center for Psychosocial Medicine, University Medical Center Hamburg-Eppendorf.

## Declarations

### Generative AI and AI-assisted technologies in the writing process

During the preparation of this work, the authors used ChatGPT-3.5 in order to improve readability and language of the work. After using this tool, the authors reviewed and edited the content as needed and take full responsibility for the content of the publication.

## Abbreviations

AVE: Average variance extracted
CB: Corinna Bergelt
CFA: Confirmatory factor analysis
CFI: Comparative fit index
CI: Confidence Interval
Df: Degrees of freedome
HCP: Healthcare professional
ICC: Intra-class Correlation Coefficient
LI: Laura Inhestern
LJ: Lene Johannsen
M: Mean
MLN: Mona L. Nasse
RMSEA: Root mean square error of approximation
SD: Standard Deviation
SE-12: Self-efficacy questionnaire
SE-12-G: Self-efficacy questionnaire, german version
SRMR: Standardized root mean square
TLI: Tucker-lewis index
TRAPD: Translation, review, adjudication, pretesting and documentation
UKE: University Medical Center Hamburg-Eppendorf
WF: Wiebke Frerichs

## References

[1] Moore PM, Rivera S, Bravo-Soto GA, Olivares C, Lawrie TA. Communication skills training for healthcare professionals working with people who have cancer. Cochrane Database Syst Rev. 2018;7(7):Cd003751. 30039853 10.1002/14651858.CD003751.pub4 PMC6513291

[2] Kissane DW, Bylund CL, Banerjee SC, Bialer PA, Levin TT, Maloney EK, D’Agostino TA. Communication skills training for oncology professionals. J Clin Oncol. 2012;30(11):1242–7. 22412145 10.1200/JCO.2011.39.6184 PMC3341141

[3] Stewart MA. Effective physician-patient communication and health outcomes: a review. CMAJ. 1995;152(9):1423–33. 7728691 PMC1337906

[4] Mauksch LB, Dugdale DC, Dodson S, Epstein R. Relationship, communication, and efficiency in the medical encounter: creating a clinical model from a literature review. Arch Intern Med. 2008;168(13):1387–95. 18625918 10.1001/archinte.168.13.1387

[5] Street RL Jr., Makoul G, Arora NK, Epstein RM. How does communication heal? Pathways linking clinician-patient communication to health outcomes. Patient Educ Couns. 2009;74(3):295–301. 19150199 10.1016/j.pec.2008.11.015

[6] Williams S, Weinman J, Dale J. Doctor-patient communication and patient satisfaction: a review. Fam Pract. 1998;15(5):480–92. 9848436 10.1093/fampra/15.5.480

[7] Stewart M, Brown JB, Donner A, McWhinney IR, Oates J, Weston WW, Jordan J. The impact of patient-centered care on outcomes. J Fam Pract. 2000;49(9):796–804. 11032203

[8] Fallowfield L, Jenkins V. Effective communication skills are the key to good cancer care. Eur J Cancer. 1999;35(11):1592–7. 10673967 10.1016/s0959-8049(99)00212-9

[9] Harrington J, Noble LM, Newman SP. Improving patients’ communication with doctors: a systematic review of intervention studies. Patient Educ Couns. 2004;52(1):7–16. 14729285 10.1016/s0738-3991(03)00017-x

[10] Uitterhoeve RJ, Bensing JM, Grol RP, Demulder PH, T VANA. The effect of communication skills training on patient outcomes in cancer care: a systematic review of the literature. Eur J Cancer Care (Engl). 2010;19(4):442–57. 20030702 10.1111/j.1365-2354.2009.01082.x

[11] Kerr D, Ostaszkiewicz J, Dunning T, Martin P. The effectiveness of training interventions on nurses’ communication skills: A systematic review. Nurse Educ Today. 2020;89:104405. 32244125 10.1016/j.nedt.2020.104405

[12] de Mata ÁNdS KPM, Braga LP, de Medeiros GCBS, de Oliveira Segundo VH, Bezerra INM, et al. Training in communication skills for self-efficacy of health professionals: a systematic review. Hum Resour Health. 2021;19(1):30. 33676515 10.1186/s12960-021-00574-3 PMC7937280

[13] Berg MN, Ngune I, Schofield P, Grech L, Juraskova I, Strasser M, et al. Effectiveness of online communication skills training for cancer and palliative care health professionals: A systematic review. Psychooncology. 2021;30(9):1405–19. 33909328 10.1002/pon.5702

[14] Frerichs W, Geertz W, Johannsen LM, Inhestern L, Bergelt C. Child- and family-specific communication skills trainings for healthcare professionals caring for families with parental cancer: A systematic review. PLoS ONE. 2022;17(11):e0277225. 36350839 10.1371/journal.pone.0277225 PMC9645618

[15] Axboe MK, Christensen KS, Kofoed PE, Ammentorp J. Development and validation of a self-efficacy questionnaire (SE-12) measuring the clinical communication skills of health care professionals. BMC Med Educ. 2016;16(1):272. 27756291 10.1186/s12909-016-0798-7 PMC5069791

[16] Efthymiou A, Kalaitzaki A, Rovithis M. Validation of the Self-Efficacy questionnaire (SE-12-Gr) assessing the healthcare professionals’ Self-Reported communication skills with older healthcare users in Greece. Health Commun. 2024;40(3):481–91. 10.1080/10410236.2024.2348841 10.1080/10410236.2024.2348841 38711248

[17] Gil C-r. Validity and reliability of the Korean version of Self-Efficacy Questionnaire(KSE-12). J Digit Convergence. 2020;18:337–45.

[18] Escribano S, Juliá-Sanchis R, García-Sanjuán S, Congost-Maestre N, Cabañero-Martínez MJ. Psychometric properties of the attitudes towards medical communication scale in nursing students. Peer J. 2021;9:e11034. 34113481 10.7717/peerj.11034 PMC8162233

[19] Wolderslund M, Kofoed PE, Ammentorp J. The effectiveness of a person-centred communication skills training programme for the health care professionals of a large hospital in Denmark. Patient Educ Couns. 2021;104(6):1423–30. 33303282 10.1016/j.pec.2020.11.018

[20] Hvidt EA, Ammentorp J, Søndergaard J, Timmermann C, Hansen DG, Hvidt NC. Developing and evaluating a course programme to enhance existential communication with cancer patients in general practice. Scand J Prim Health Care. 2018;36(2):142–51. 29623752 10.1080/02813432.2018.1459235 PMC6066852

[21] Desai S, Chen F, Boynton-Jarrett R. Clinician satisfaction and Self-Efficacy with centeringparenting group Well-Child care model: A pilot study. J Prim Care Community Health. 2019;10:2150132719876739. 31550973 10.1177/2150132719876739 PMC6764027

[22] Kk A, At J, Lø P, Jd L. Effects of on-site supportive communication training (On-site SCT) on doctor-patient communication in oncology: study protocol of a randomized, controlled mixed-methods trial. BMC Med Educ. 2024;24(1):522. 38730382 10.1186/s12909-024-05496-x PMC11088166

[23] Johannsen LM, Frerichs W, Philipp R, Inhestern L, Bergelt C. Effectiveness of a training program for healthcare professionals on parental cancer: results of a randomized controlled pilot-study. Psycho-oncology. 2023;32(10):1567–77. 37649177 10.1002/pon.6207

[24] Inhestern L, Frerichs W, Johannsen LM, Bergelt C. Process-evaluation and outcome-evaluation of a training programme for healthcare professionals in oncology to enhance their competencies in caring for patients with minor children: a study protocol for a randomised controlled pilot study. BMJ Open. 2019;9(10):e032778. 10.1136/bmjopen-2019-032778 PMC6797360 31615803

[25] Harkness JA, Villar A, Edwards B. Translation, adaptation, and design. Survey methods in multinational, multiregional, and multicultural contexts. Wiley series in survey methodology. Hoboken, NJ, US: John Wiley & Sons, Inc. 2010;117–40.

[26] Mohler P, Dorer B, De Jong J, Hu M. Translation. Guidelines for best practice in cross-cultural surveys. Ann Arbor, MI: survey research center Institute for social research University of Michigan 2016.

[27] Harkness J, Pennell B-E, Schoua-Glusberg A. Survey Questionnaire Translation and Assessment. Methods for Testing and Evaluating Survey Questionnaires 2004;453– 73.

[28] Prüfer P, Rexroth M, Kognitive Interviews. 2005 [Available from: https://www.gesis.org/fileadmin/upload/forschung/publikationen/gesis_reihen/hohow/How_to15PP_MR.pdf

[29] Bannon W. Jr. Missing data within a quantitative research study: how to assess it, treat it, and why you should care. J Am Assoc Nurse Pract. 2015;27(4):230–2. 25676704 10.1002/2327-6924.12208

[30] Flake JK, Pek J, Hehman E. Construct validation in social and personality research:current practice and recommendations. Social Psychol Personality Sci. 2017;8(4):370–8.

[31] Flora DB, Flake JK. The purpose and practice of exploratory and confirmatory factor analysis in psychological research: decisions for scale development and validation. Can J Behav Sci / Revue Canadienne Des Sci Du Comportement. 2017;49(2):78–88.

[32] Rogers P. Best practices for your exploratory factor analysis: A factor tutorial. J Contemp Adm. 2021;26(6).

[33] Rattray J, Jones MC. Essential elements of questionnaire design and development. J Clin Nurs. 2007;16(2):234–43. 10.1111/j.1365-2702.2006.01573.x 17239058

[34] Streiner DL, Norman GR, Cairney J. Health measurement scales: A practical guide to their development and use. Oxford University Press 2014. 2015.

[35] Enders CK, Bandalos DL. The relative performance of full information maximum likelihood Estimation for missing data in structural equation models. Struct Equation Modeling: Multidisciplinary J. 2001;8(3):430–57.

[36] Li C-H. Confirmatory factor analysis with ordinal data: comparing robust maximum likelihood and diagonally weighted least squares. Behav Res Methods. 2016;48(3):936–49. 26174714 10.3758/s13428-015-0619-7

[37] Lt H, Bentler PM. Cutoff criteria for fit indexes in covariance structure analysis: conventional criteria versus new alternatives. Struct Equation Modeling: Multidisciplinary J. 1999;6(1):1–55.

[38] Hair JBW, Babin B, Anderson RE. Multivariate data analysis. Upper Saddle River, NJ: Pearson Prentice Hall 2009.

[39] Ferketich S. Focus on psychometrics. Aspects of item analysis. Res Nurs Health. 1991;14(2):165–8. 2047538 10.1002/nur.4770140211

[40] Boateng GO, Neilands TB, Frongillo EA, Melgar-Quiñonez HR, Young SL. Best practices for developing and validating scales for health, social, and behavioral research: A primer. Front Public Health. 2018;6. 10.3389/fpubh.2018.00149 PMC6004510 29942800

[41] McHorney CA, Tarlov AR. Individual-patient monitoring in clinical practice: are available health status surveys adequate? Qual Life Res. 1995;4(4):293–307. 7550178 10.1007/BF01593882

[42] Bannigan K, Watson R. Reliability and validity in a nutshell. J Clin Nurs. 2009;18(23):3237–43. 19930083 10.1111/j.1365-2702.2009.02939.x

[43] Hooper D, Coughlan J, Mullen MR. Structural equation modelling: guidelines for determining model fit. Electron J Bus Res Methods. 2008;6:53–60.

[44] Browne MW, Cudeck R. Single sample Cross-Validation indices for covariance structures. Multivar Behav Res. 1989;24(4):445–55. 10.1207/s15327906mbr2404_4 26753509

[45] Fornell C, Larcker DF. Evaluating structural equation models with unobservable variables and measurement error. J Mark Res. 1981;18(1):39–50.

[46] Cheung GW, Cooper-Thomas HD, Lau RS, Wang LC. Reporting reliability, convergent and discriminant validity with structural equation modeling: A review and best-practice recommendations. Asia Pac J Manage. 2024;41(2):745–83.

[47] Marsh HW, Hau KT. Confirmatory Factor Analysis: Strategies for Small Sample Sizes. Statistical Strategies for Small Sample Research. Statistical Strategies for Small Sample Research. 1999;1:251– 84.

[48] Portney LG, Watkins MP. Foundations of clinical research: applications to practice. Pearson/Prentice Hall 2015.

[49] Matheson GJ. We need to talk about reliability: making better use of test-retest studies for study design and interpretation. PeerJ. 2019;7:e6918. 31179173 10.7717/peerj.6918 PMC6536112

[50] Shiu E, Pervan SJ, Bove LL, Beatty SE. Reflections on discriminant validity: reexamining the Bove et al. (2009) findings. J Bus Res. 2011;64(5):497–500.

[51] Kyriazos TA. Applied psychometrics: sample size and sample power considerations in factor analysis (EFA, CFA) and SEM in general. Psychology. 2018;l09(08):25.

