# Supplementary material for: The German version of the self-efficacy questionnaire (SE-12-G) for measuring clinical communication skills in a sample of healthcare professionals: translation and psychometric properties
Source: BMC Med Educ. 2025 Jul 17;25:1069. doi: 10.1186/s12909-025-07681-y (PMC12273301; doi:10.1186/s12909-025-07681-y)
Supplement: Supplementary file 1 — Supplementary Material 1 [file 12909_2025_7681_MOESM1_ESM.pdf]

Additional file 1: SE-12-G

Kommunikation mit Patientinnen und Patienten

Die folgenden Fragen beziehen sich auf ausgewählte Fähigkeiten in der Kommunikation mit Patientinnen und Patienten.
Bitte beantworten Sie jede Frage.
Sofern eine Frage Ihren beruflichen Alltag nicht betrifft, kreuzen Sie bitte „*nicht zutreffend*“ an.

SE_Y_to

Wie sicher sind Sie darin, …

1a	…die Anliegen zu erkennen, die eine Patientin oder ein Patient in einem Gespräch ansprechen möchte?	sehr unsicher ❑	❑	❑	❑	❑	❑	❑	❑	❑	sehr sicher ❑	nicht zutreffend ❑
1b	*Für wie wichtig halten Sie es, dies im beruflichen Alltag umzusetzen?*	sehr unwichtig ❑	eher unwichtig ❑	teils/teils ❑	eher wichtig ❑	sehr wichtig ❑						
2a	…die Themen für das Gespräch mit der Patientin oder dem Patienten gemeinsam festzulegen?	sehr unsicher ❑	❑	❑	❑	❑	❑	❑	❑	❑	sehr sicher ❑	nicht zutreffend ❑
2b	*Für wie wichtig halten Sie es, dies im beruflichen Alltag umzusetzen?*	sehr unwichtig ❑	eher unwichtig ❑	teils/teils ❑	eher wichtig ❑	sehr wichtig ❑						
3a	…eine Patientin oder einen Patienten zu ermutigen, ihre oder seine Probleme/Sorgen auszudrücken und zu besprechen?	sehr unsicher ❑	❑	❑	❑	❑	❑	❑	❑	❑	sehr sicher ❑	nicht zutreffend ❑
3b	*Für wie wichtig halten Sie es, dies im beruflichen Alltag umzusetzen?*	sehr unwichtig ❑	eher unwichtig ❑	teils/teils ❑	eher wichtig ❑	sehr wichtig ❑						
4a	…einer Patientin oder einem Patienten aufmerksam zuzuhören, ohne zu unterbrechen oder das Thema zu wechseln?	sehr unsicher ❑	❑	❑	❑	❑	❑	❑	❑	❑	sehr sicher ❑	nicht zutreffend ❑
4b	*Für wie wichtig halten Sie es, dies im beruflichen Alltag umzusetzen?*	sehr unwichtig ❑	eher unwichtig ❑	teils/teils ❑	eher wichtig ❑	sehr wichtig ❑						
5a	…eine Patientin oder einen Patienten zu ermutigen, ihre oder seine Gedanken und Gefühle auszudrücken?	sehr unsicher ❑	❑	❑	❑	❑	❑	❑	❑	❑	sehr sicher ❑	nicht zutreffend ❑
5b	*Für wie wichtig halten Sie es, dies im beruflichen Alltag umzusetzen?*	sehr unwichtig ❑	eher unwichtig ❑	teils/teils ❑	eher wichtig ❑	sehr wichtig ❑						
6a	… ein Gespräch mit Patientinnen und Patienten zu strukturieren?	sehr unsicher ❑	❑	❑	❑	❑	❑	❑	❑	❑	sehr sicher ❑	nicht zutreffend ❑
6b	*Für wie wichtig halten Sie es, dies im beruflichen Alltag umzusetzen?*	sehr unwichtig ❑	eher unwichtig ❑	teils/teils ❑	eher wichtig ❑	sehr wichtig ❑						

Wie sicher sind Sie darin, …

Nr.	Frage											
7a	… angemessenes non-verbales Verhalten im Patient:innengespräch zu zeigen (z.B. Blickkontakt, Mimik, Stimmlage, Körperhaltung)?	sehr unsicher ❑	❑	❑	❑	❑	❑	❑	❑	❑	sehr sicher ❑	nicht zutreffend ❑
7b	*Für wie wichtig halten Sie es, dies im beruflichen Alltag umzusetzen?*	sehr unwichtig ❑	eher unwichtig ❑	teils/teils ❑	eher wichtig ❑	sehr wichtig ❑						
8a	… Einfühlungsvermögen (Empathie) zu zeigen (z.B. in Bezug auf die Ansichten und Gefühle der Patientin oder des Patienten)?	sehr unsicher ❑	❑	❑	❑	❑	❑	❑	❑	❑	sehr sicher ❑	nicht zutreffend ❑
8b	*Für wie wichtig halten Sie es, dies im beruflichen Alltag umzusetzen?*	sehr unwichtig ❑	eher unwichtig ❑	teils/teils ❑	eher wichtig ❑	sehr wichtig ❑						
9a	… zu klären, welchen Wissensstand die Patientin oder der Patient hat, um eine angemessene Menge an Informationen zu vermitteln?	sehr unsicher ❑	❑	❑	❑	❑	❑	❑	❑	❑	sehr sicher ❑	nicht zutreffend ❑
9b	*Für wie wichtig halten Sie es, dies im beruflichen Alltag umzusetzen?*	sehr unwichtig ❑	eher unwichtig ❑	teils/teils ❑	eher wichtig ❑	sehr wichtig ❑						
10a	… zu erfragen, inwieweit die Patientin oder der Patient die vermittelten Informationen verstanden hat?	sehr unsicher ❑	❑	❑	❑	❑	❑	❑	❑	❑	sehr sicher ❑	nicht zutreffend ❑
10b	*Für wie wichtig halten Sie es, dies im beruflichen Alltag umzusetzen?*	sehr unwichtig ❑	eher unwichtig ❑	teils/teils ❑	eher wichtig ❑	sehr wichtig ❑						
11a	… einen Plan für das weitere Vorgehen auf Basis der gemeinsam getroffenen Entscheidungen zwischen Ihnen und der Patientin oder dem Patienten zu erstellen?	sehr unsicher ❑	❑	❑	❑	❑	❑	❑	❑	❑	sehr sicher ❑	nicht zutreffend ❑
11b	*Für wie wichtig halten Sie es, dies im beruflichen Alltag umzusetzen?*	sehr unwichtig ❑	eher unwichtig ❑	teils/teils ❑	eher wichtig ❑	sehr wichtig ❑						
12a	… ein Gespräch mit Patientinnen und Patienten zu beenden und dabei sicherzustellen, dass alle Fragen der Patientin oder des Patienten beantwortet wurden?	sehr unsicher ❑	❑	❑	❑	❑	❑	❑	❑	❑	sehr sicher ❑	nicht zutreffend ❑
12b	*Für wie wichtig halten Sie es, dies im beruflichen Alltag umzusetzen?*	sehr unwichtig ❑	eher unwichtig ❑	teils/teils ❑	eher wichtig ❑	sehr wichtig ❑						
