# Supplementary material for: The German version of the self-efficacy questionnaire (SE-12-G) for measuring clinical communication skills in a sample of healthcare professionals: translation and psychometric properties
Source: BMC Med Educ. 2025 Jul 17;25:1069. doi: 10.1186/s12909-025-07681-y (PMC12273301; doi:10.1186/s12909-025-07681-y)

**Confirmatory Factor Analysis - Model 1**


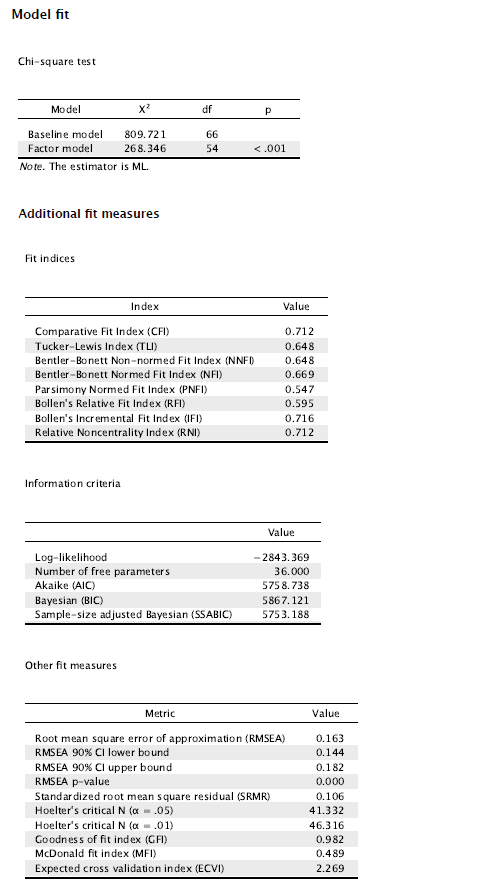


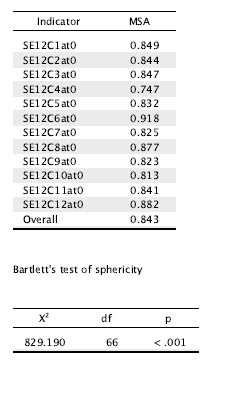


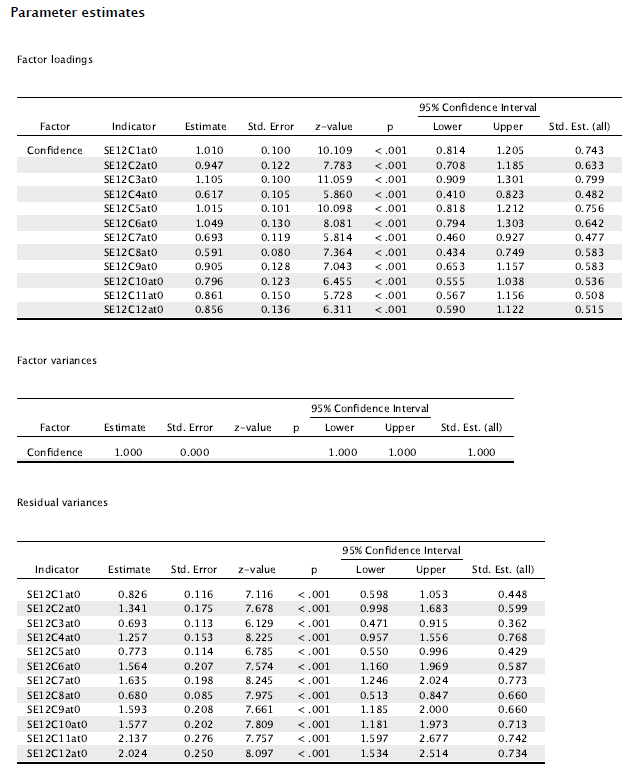


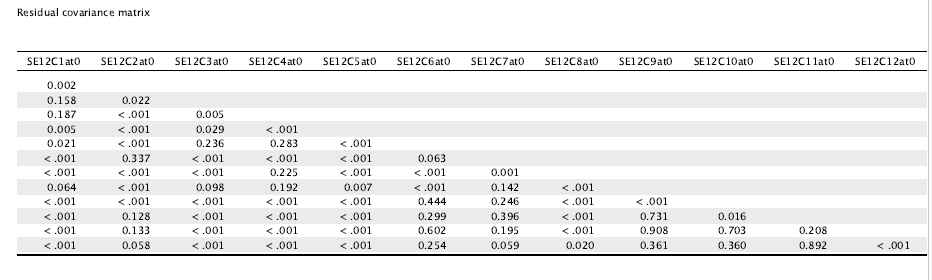


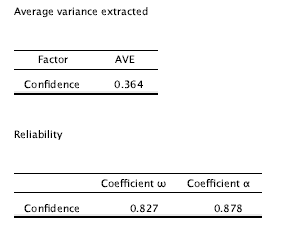


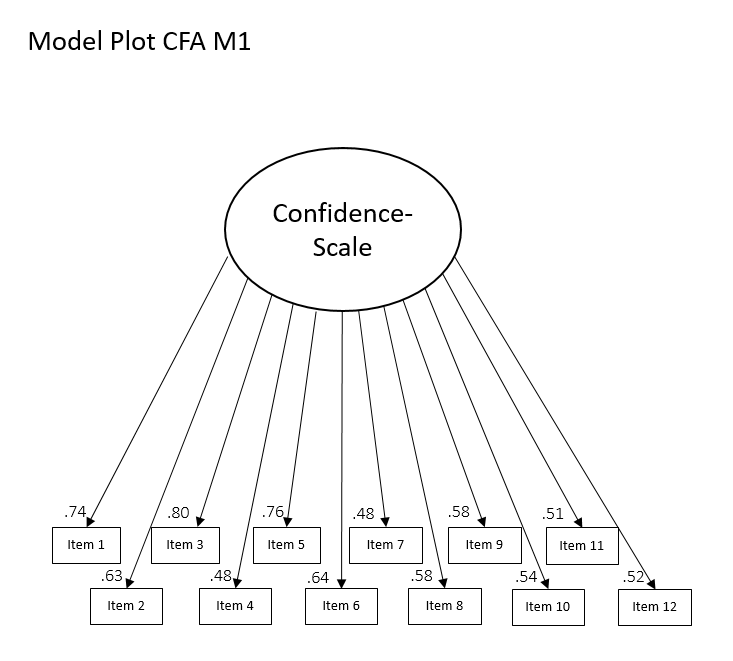


**Confirmatory Factor Analysis - Model 2**


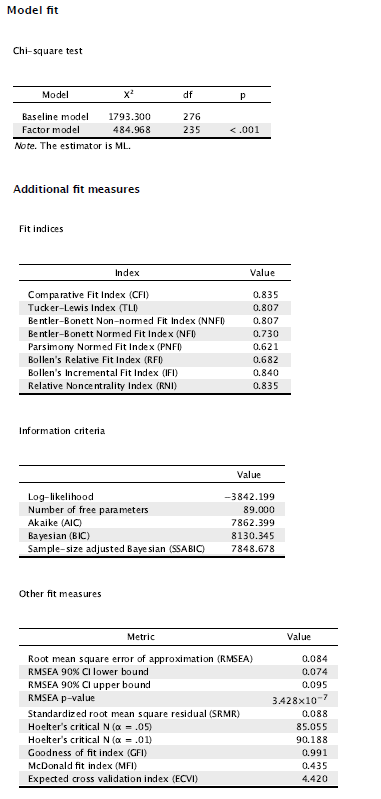


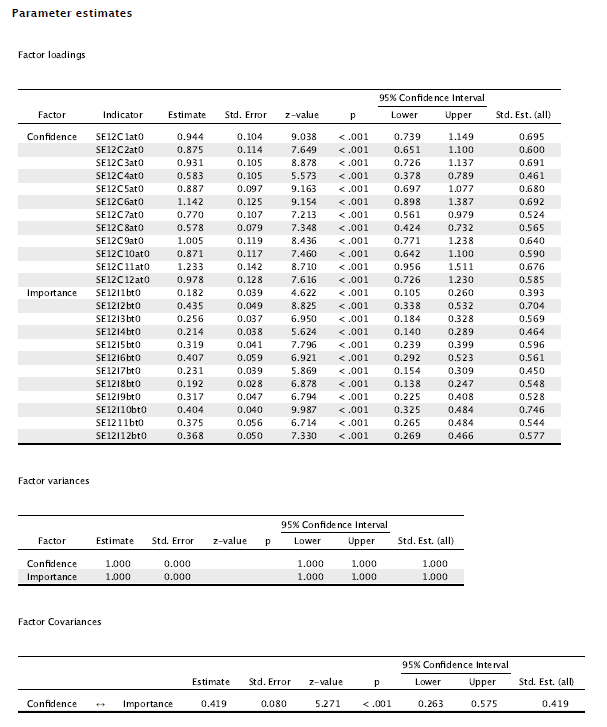


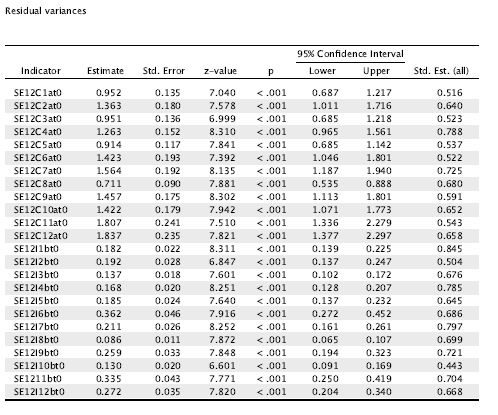


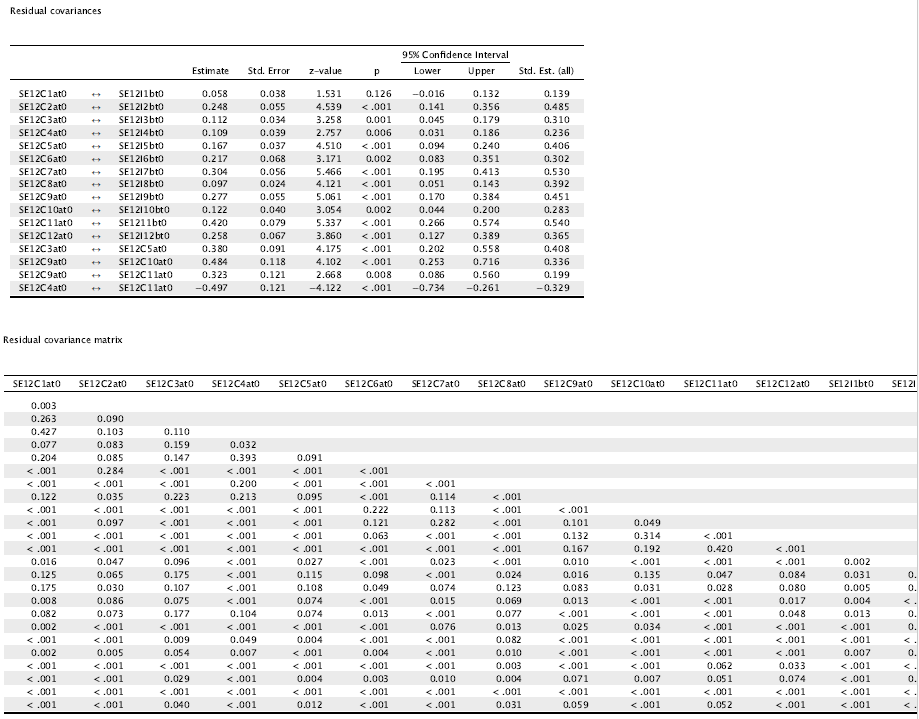


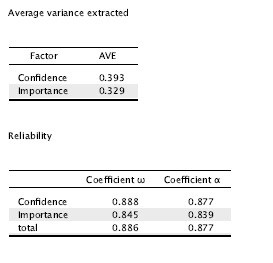


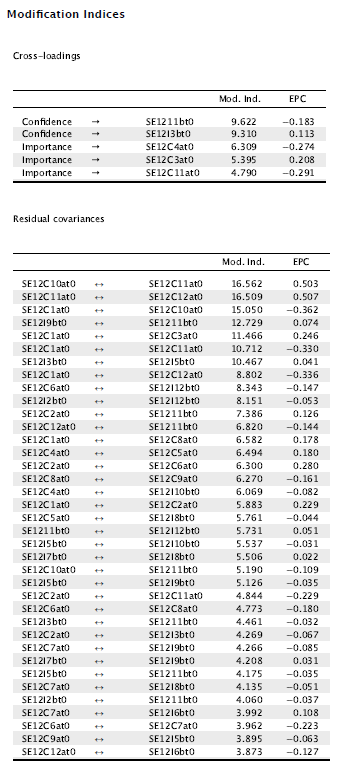


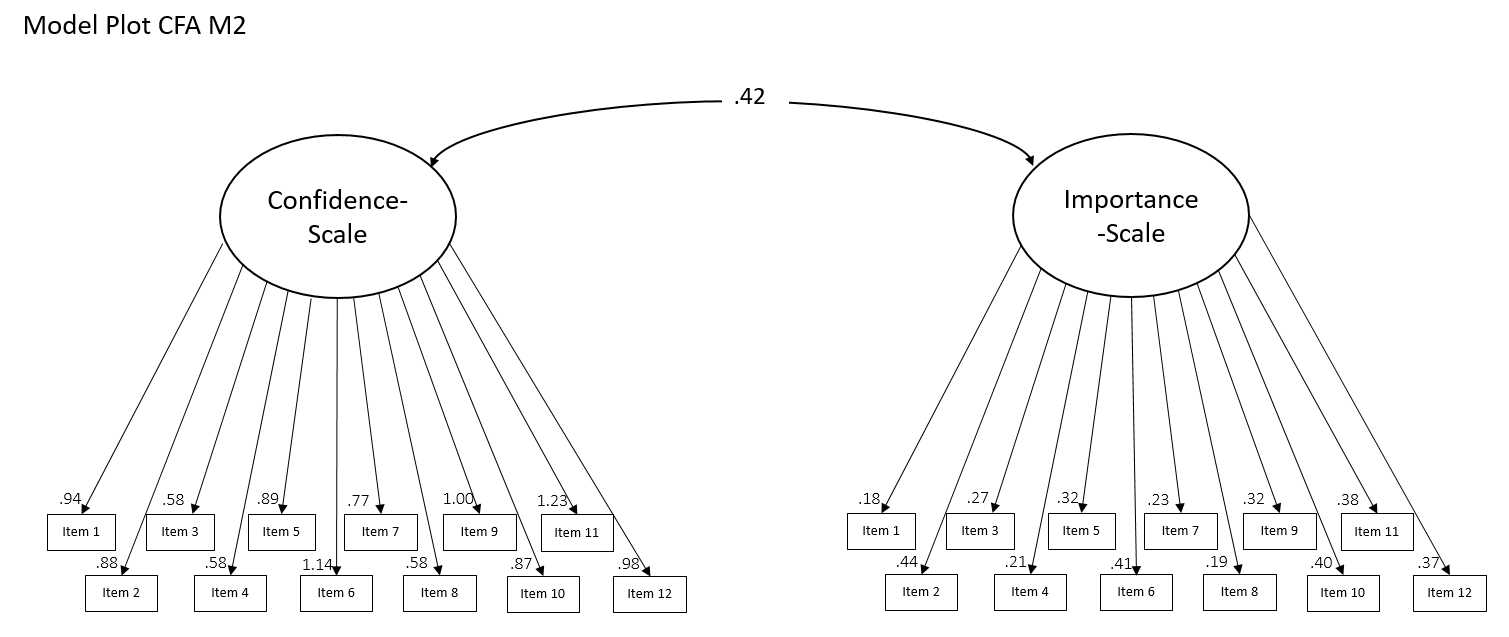


**Confirmatory Factor Analysis - Model 3a**


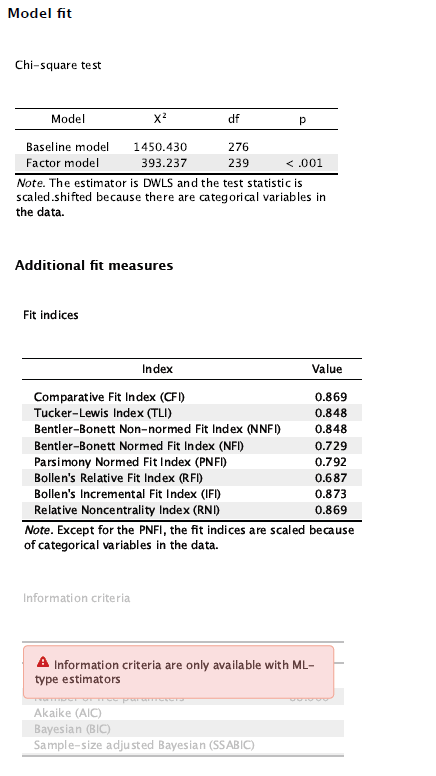


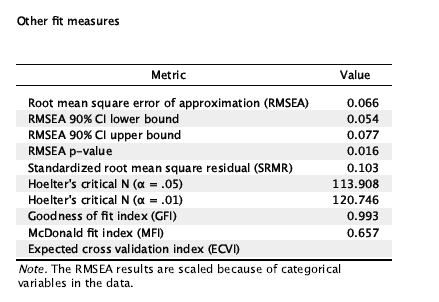


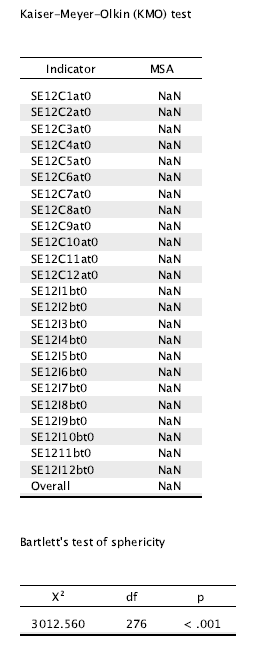


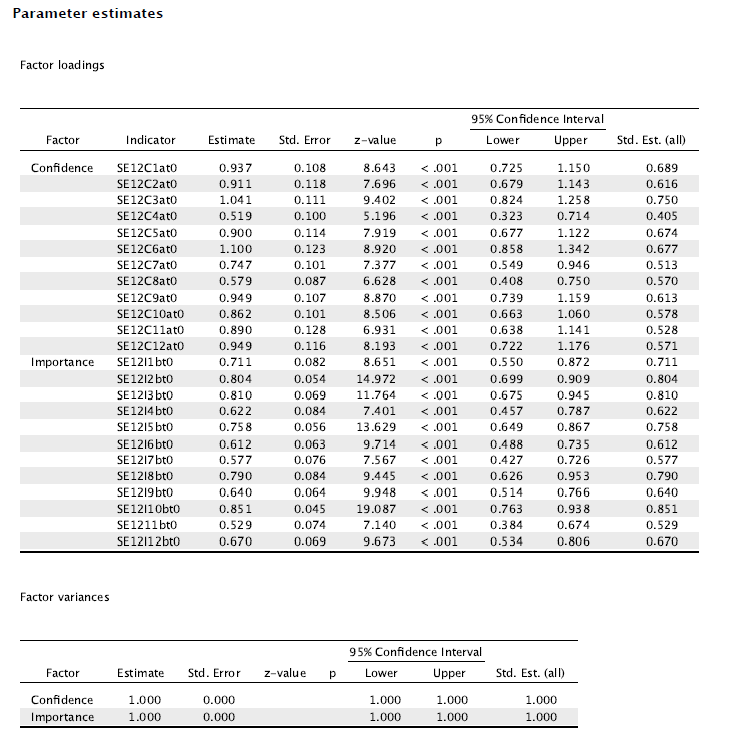


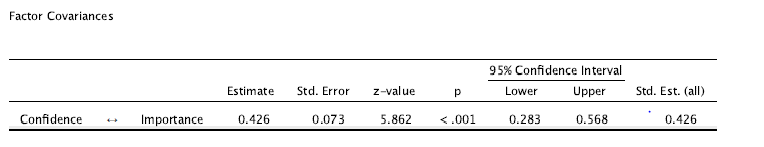


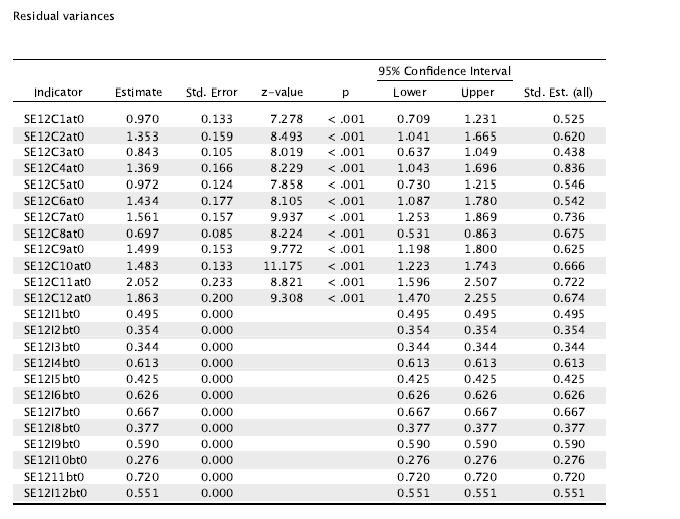


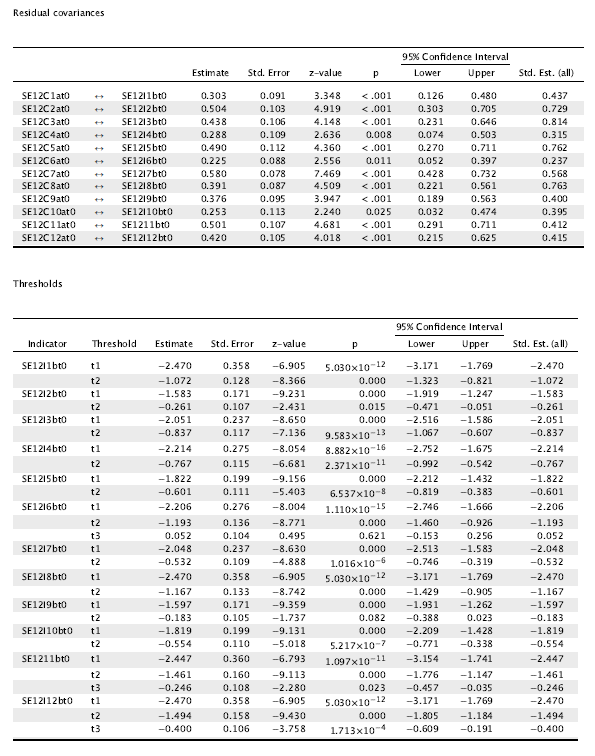


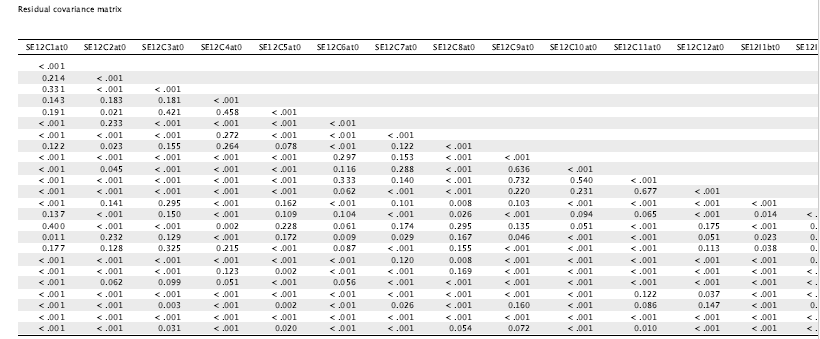


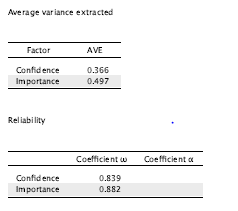


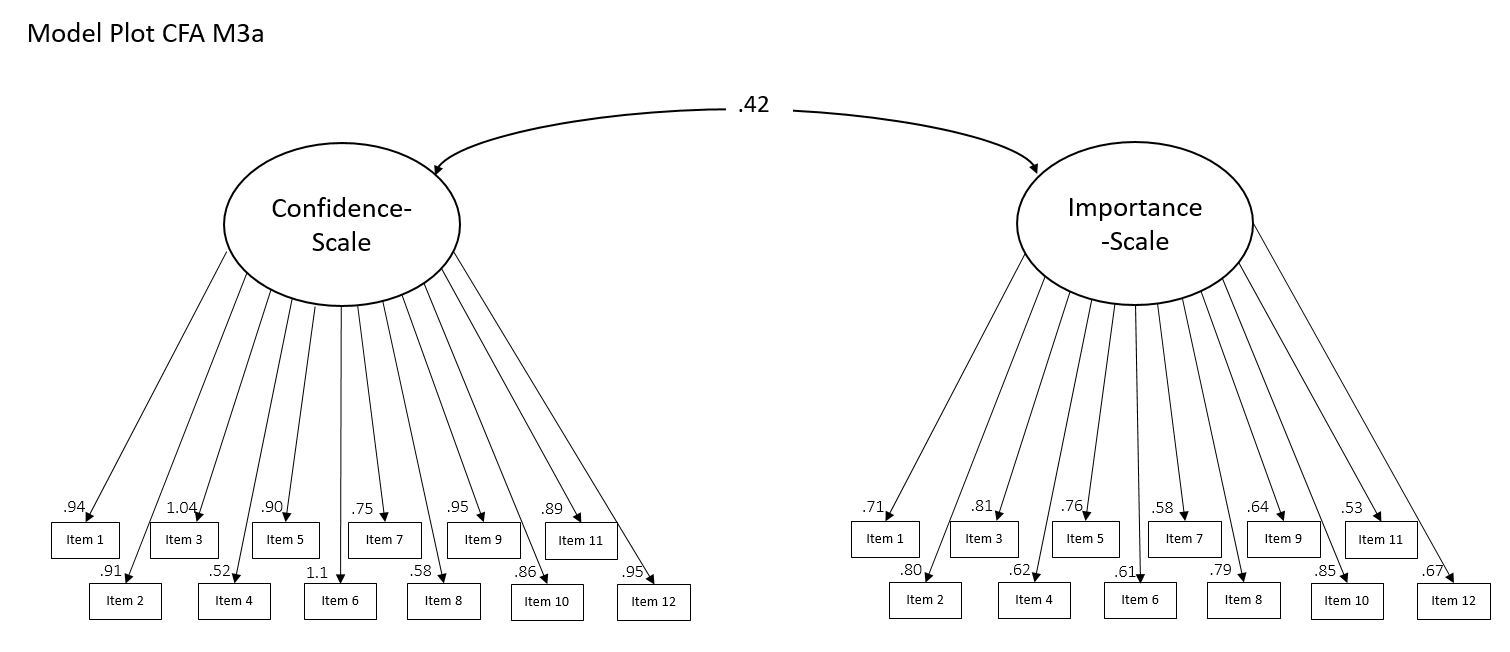


Model CFA M3b:


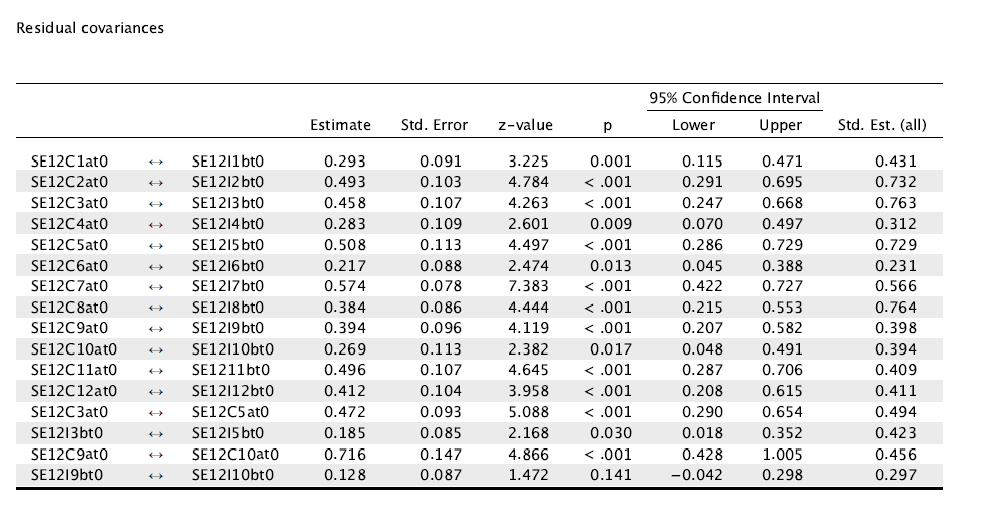

Supplement: Supplementary file 2 — Supplementary Material 2 [file 12909_2025_7681_MOESM2_ESM.docx]
